# Malnutrition, nutrition support and dietary intervention: the role of the dietitian supporting patients with head and neck cancer

**DOI:** 10.1038/s41415-022-5107-8

**Published:** 2022-11-11

**Authors:** Florence Cook, Jose M. Rodriguez, Lorna K. McCaul

**Affiliations:** 4141556233001 Head and Neck Oncology Dietitian, Department of Nutrition and Dietetics, University College London Hospital NHS Foundation Trust, London, UK; 4141556233002 Consultant in Restorative Dentistry and Honorary Clinical Senior Lecturer, Faculty of Dentistry, Oral and Craniofacial Sciences, King´s College London, 26th Floor, Tower Wing, Great Maze Pond, SE1 9RT, London, UK; 4141556233003 Consultant and Honorary Clinical Senior Lecturer in Restorative Dentistry, Department of Restorative Dentistry, Glasgow Dental Hospital and School, NHS Greater Glasgow and Clyde, Glasgow, Scotland, UK

## Abstract

Malnutrition is prevalent in patients with head and neck cancer (HNC) at diagnosis but can occur at any stage of the treatment pathway. The impact of disease burden and treatment side effects can lead to altered anatomy, compromised quality and quantity of saliva and impaired swallowing function, which can result in deleterious effects on nutritional status. Optimising nutrition status is critical, as malnutrition is adversely associated with treatment tolerance and outcomes, wound healing, morbidity, mortality, quality of life and survival. Dietitians are integral members of the HNC multidisciplinary team and are uniquely qualified in the assessment, management and optimisation of nutritional status across the care pathway. This includes providing informational counselling to patients and carers on the short- and long-term nutritional impact of planned treatments alongside multidisciplinary members. Dietitians lead on the recommendation, provision and monitoring of nutrition support, which can be via the oral, enteral or parenteral route. Oral nutrition support includes dietary counselling, nourishing dietary, food fortification advice and high energy/protein oral nutritional supplements. Enteral nutrition support, or tube feeding, can be required on a short- and/or long-term basis and dietitians support appropriate decision-making for the type of tube and timing of placement across the care pathway.

## Key points

- Head and neck cancer (HNC) is a multifaceted disease requiring multi-disciplinary intervention. The impact of tumour burden and treatment side effects can lead to altered anatomy, impaired swallowing function and deleterious effects on nutritional status. Many patients are at risk of malnutrition which can occur at any stage of the care pathway.
- Dietitians are core members of the HNC multi-disciplinary team and lead on the nutritional management of patients at any stage of the treatment pathway, from diagnosis until death, palliation and/or survivorship.
- Nutrition support is vital in the prevention and treatment of malnutrition. This includes dietary counselling, nourishing dietary advice and the provision of high energy/protein oral nutritional supplements, as well as enteral tube feeding on a short- and/or long-term basis when indicated. Dietitians work alongside restorative dentists to balance nutritional needs with prevention of dental caries.

## Introduction

Head and neck cancer (HNC) is multifaceted in nature. The aetiology of the disease and consequences of treatment have a profound impact on nutritional status and risk of malnutrition. This includes impact on functional swallowing status and the ability to take adequate nutrition orally and/or via alternative nutrition support. Nutritional intervention is integral in preventing disease- and treatment-related malnutrition and/or weight loss. Dietitians are healthcare professionals that are uniquely qualified to translate scientific information about food and nutrition into practical dietary advice. Within HNC, specialist dietitians are integral members of the multidisciplinary team (MDT) and lead on assessing patients' nutritional status and needs, alongside providing individualised nutritional advice. This often includes the provision of nutritional support throughout the patient's journey, from diagnosis to survivorship, palliation and/or death.^1^

## Malnutrition and survival

Patients with HNC are at a high risk of malnutrition which takes place when a negative energy balance occurs due to reduced intake or uptake of nutrients, resulting in unintentional weight loss.^2^ This can occur at any stage of the patient pathway and has been reported in 60% of HNC patients before commencing treatment,^3^ increasing to up to 86% at the end of chemoradiotherapy.^4^ Malnutrition is common due to the impact of the disease site, tumour burden and side effects of treatment modalities (see Table 1).^5^^,^^6^

**Table 1:** Causes of malnutrition in HNC^5^^,^^6^

Cause	Impact
Tumour characteristics and disease	Mechanical issues with chewing and altered anatomy obstructing any/all stages of swallowing, which can result in: Dysphagia (difficulty in swallowing) Odynophagia (pain when swallowing) Aspiration (passage of food or fluids into the lungs) Limitations on dietary textures tolerated Metabolic: Increased nutritional requirements due to tumour burden Reduced appetite Cachexia (unintentional weight loss, loss of appetite and muscle wasting) Sarcopenia (loss of muscle mass and strength)
Treatment: surgery	Dysphagia Reduced appetite (for example, nausea post anaesthesia) Increased nutritional requirements to support recovery, wound healing and any infection Potential periods of pre-procedure fasting due to investigations leading to deficits in energy intake Resection leading to altered anatomy and impaired swallowing function, affecting ability to take nutrition orally, often requiring enteral nutrition support
Treatment: radiotherapy	Dysphagia Odynophagia Aspiration Dysgeusia (altered taste sensation) Pain Fatigue Mucositis (painful ulceration and inflammation of mucosal membranes) Xerostomia (dry mouth) Hyposalivation Osteoradionecrosis (exposed irradiated bone that fails to heal over three months without evidence of recurrent or persistent tumour)^46^
Treatment: chemotherapy	Dysgeusia Dysphagia Mucositis Poor appetite Gastrointestinal leading to malabsorption: Nausea and vomiting Diarrhoea Anorexia (loss of appetite) Fatigue
Social	Poor access to food and drink Poor baseline dietary patterns/habits and choices High intake of alcohol Lack of social support Depression Restricting dietary choices and pursuing popular 'fad' diets (for example, the alkaline diet) due to media misrepresentation and pseudo-scientific rationales for anti-cancer properties. These have limited evidence to suggest that they improve cancer outcomes and can adversely affect nutritional status and exacerbate malnutrition^30^^,^^47^
Dental	Reduction in numbers of masticatory units due to surgical resection of tooth bearing jawbone or pre-treatment extractions resulting in difficulties with comminution Inability to wear dentures due to mucosal changes or altered anatomy

Recent studies have reported that patients who are overweight/obese at presentation can be at a higher risk of becoming malnourished during treatment.^7^ Malnutrition is adversely associated with treatment tolerance, wound healing, surgical complications, morbidity, mortality, quality of life (QofL) and survival.^6^ Furthermore, weight loss is a predictor for poor survival and treatment tolerance.^8^ All MDT members can support screening for malnutrition and refer to the dietitian for assessment and intervention at the earliest opportunity to prevent further decline.^9^

Cachexia in cancer is a complex disease characterised by unintentional weight loss, loss of appetite and muscle wasting. Cachexia differs from conventional starvation where lean muscle is preserved and adipose tissue is mainly affected. It is associated with metabolic abnormalities leading to an overall catabolic state and systemic inflammation. Prevalence in HNC can range from 6.1% at diagnosis to 41% post treatment.^10^ It is challenging to manage, as aggressive nutritional intervention is insufficient to reverse and/or prevent further loss, leading to functional impairment.^11^

Beyond weight and body mass index, dietitians can also undertake an assessment of body composition to analyse the nature of overall weight loss and differentiate between loss of adipose tissue and loss of skeletal muscle. Commonly used methods include measurement of hand-grip strength, bioelectrical impedance analysis and skinfold thickness. When accessible, dual-energy x-ray absorptiometry and computer tomography can be used for in-depth body composition assessment.^12^ Sarcopenia is a condition characterised as the loss of muscle mass and strength and is associated with reduced survival. Prevalence in HNC has been reported to range from 6.6-64.6% before commencing radiotherapy with curative intent ± other treatment modalities increasing to 12.4-65.8% post-treatment.^13^

## Impact of the dietitian and nutritional counselling

Specialist dietitians are integral members of the HNC MDT and provide individualised nutritional advice throughout the patient's continuum of care. UK standards state that all HNC units should have a specialist dietitian with at least 50% of their clinical time dedicated to HNC.^1^ Nutritional counselling has an important role to play in the management of HNC, with positive effects reported on nutritional intake, nutritional status, clinical outcomes and QofL.^3^ Individualised nutritional counselling given by a dietitian has been reported as more efficacious compared to generalised advice given by nursing staff or no counselling.^3^^,^^14^^,^^15^^,^^16^ Furthermore, behaviour change training is a key skill of clinicians working with patients with complex needs. Dietitians are often supported with undertaking advanced communication skills courses. The effectiveness of incorporating psychological intervention to nutritional care is gaining momentum. In Australia, the Eating As Treatment trial investigated the use of a dietitian delivered behaviour change intervention, with the goal of reducing malnutrition in HNC patients undergoing radiotherapy. Patients receiving the intervention had shorter and fewer unplanned hospital admissions alongside statistically significantly better nutritional status scores.^17^

Dietitians can also extend their scope of practice in the HNC MDT and train to become advanced clinical practitioners.^18^^,^^19^ This may include training to place enteral feeding tubes, prehabilitation programmes and undertaking post-graduate courses to become a non-medical supplementary prescriber (NMP).^19^ Dietitians are well-placed to become NMPs in the radiotherapy setting, owing to regularly reviewing patients and identifying barriers to taking adequate nutrition early. This means that dietitians can prescribe medications such as analgesia and anti-emetics to optimise tolerance of nutritional interventions.^19^

## The role of the dietitian at each point of the care pathway

**Fig. 1:**
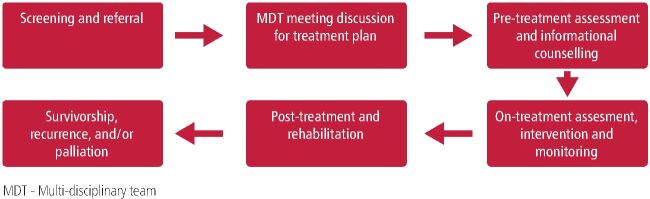
The patient care journey and the role of the dietitian at each stage

The dietitian can be referred to at any point of the care pathway (Fig. 1). The frequency of contact depends on individualised needs.^1^^,^^9^

### Screening and referral

Patients usually present initially to HNC units, where any issues with ability to meet nutritional needs are screened and identified by core MDT members. Following this, any patient identified at risk of malnutrition should be referred promptly to the dietitian for a baseline nutritional assessment and advice to prevent deterioration. This may include the provision of nutritional support to treat malnutrition via the appropriate feeding route and advice on diagnosing and treating refeeding syndrome (adverse metabolic changes that can occur when nutrition is re-introduced in those that are malnourished or in a starved state)^2^ where appropriate. It has been reported that early intervention from dietetics has been associated with improved clinical outcomes.^20^

### MDT discussion

After initial referral to the HNC unit, cases are discussed in the HNC MDT meeting. Specialist dietitians can provide input to support treatment decision-making, especially when proposed treatment plans are likely to impact on functional swallowing and nutritional status, with anticipated requirements for enteral tube feeding.

### Pre-treatment

Following the MDT meeting and decision outcomes for treatment have been made, a key role of the dietitian is to prepare the patient for the proposed treatment. All patients that are planned for treatment that will affect nutritional status should be seen in a pre-treatment appointment.^1^ The purpose of this appointment is to: provide informational counselling on the likely impact of proposed treatment on eating, drinking and nutritional status; conduct a baseline nutritional assessment (if not prior conducted); and, when applicable, provide nutritional advice and intervention to prevent decline.^9^

For patients undergoing major surgery and that are severely malnourished, this may include recommending pre-operative nutritional support with or without an admission to hospital to optimise nutritional status, as inadequate oral intake for >14 days is associated with higher mortality.^21^^,^^22^^,^^23^

For patients undergoing radiotherapy, chemoradiotherapy, or any treatment leading to enteral feeding requirements, the dietitian should discuss enteral tube feeding options. This may include prophylactic gastrostomy placement when indicated and in conjunction with locally agreed criteria and service provisions in individual units.^24^

### On-treatment

Surgery in HNC often involve resections that impact on the anatomy and physiology required for safe swallow function. Therefore, many patients require enteral feeding postoperatively on a short- or long-term basis. All patients requiring nutritional support should be reviewed by the dietitian at regular intervals during their inpatient stay. Enteral feeding, when indicated, should be initiated without delay and within 24 hours postoperatively^25^^,^^26^ as part of enhanced recovery after surgery protocols, which aim to accelerate recovery after surgery by facilitating early return to function and reduced stress through implementing multimodal care pathways.^21^ Dietitians attend ward rounds, meetings and collaborate with MDT members. For example, if/when patients can resume oral intake, dietitians liaise with the surgeons and speech and language therapists (SLTs) on the type and timing of diet that can be resumed.

Oncological treatments for HNC include radiotherapy/proton beam therapy which can be used in conjunction with surgery (adjuvant) and or chemotherapy (neoadjuvant/concurrent) or as a single-modality treatment. The side effects of radiotherapy take a cumulative effect and are detailed in Table 1.^27^ The extent of side effects impacting on the ability to take adequate oral nutrition depends on the treatment site, dose and fields. Maintaining weight stability is crucial during radiotherapy treatment due to the precise nature of mask fitting and accuracy of the treatment plan based on body measurements. Significant weight loss during treatment can result in ill-fitting masks and therefore risks treatment being stopped and/or re-planned.^28^ Therefore, any deficit should be identified early and patients should be reviewed by the dietitian at least once a week, ideally twice-weekly during treatment.^9^ Dietitians also support the MDT in encouraging adherence to treatments, such as analgesia, to alleviate side effects and in turn, optimise tolerance to nutrition support.

### Post treatment

Dietitians continue to review patients either in the acute setting or may refer to the community dietitian for patients requiring home enteral feeding. Some patients require long-term enteral feeding due to extensive treatment. Dietitians can signpost these patients to support groups and ensure feeding regimens are optimised for QofL.

The goal of dietetic intervention following treatment is to rehabilitate patients back to their baseline diet where feasible.^9^ This may include reducing the provision of enteral feeding while increasing oral intake as feasible/safe with guidance from SLTs. Side effects can be experienced on a short- and long-term basis and ongoing nutritional support may be required. Late side effects can also present, such as osteoradionecrosis, stenosis and dysphagia, which can lead to ongoing poor nutritional intake.^27^

### Palliation

Recurrence is common in HNC and patients may be referred for palliative care and/or further treatments.^29^ Nutritional status can deteriorate with disease progression and the benefits of nutrition support should account for prognosis. Dietitians work with patients, carers and MDT members to discuss, consider and balance the risks, benefits and appropriateness of initiating nutritional support, especially invasive forms, with the goal to uphold QofL and comfort.^9^^,^^30^

### Survivorship

Where patients are no longer at risk of malnutrition and as part of survivorship, dietitians can provide healthy eating and physical activity advice on an individualised basis.^9^^,^^30^

## Nutrition support

**Fig. 2:**
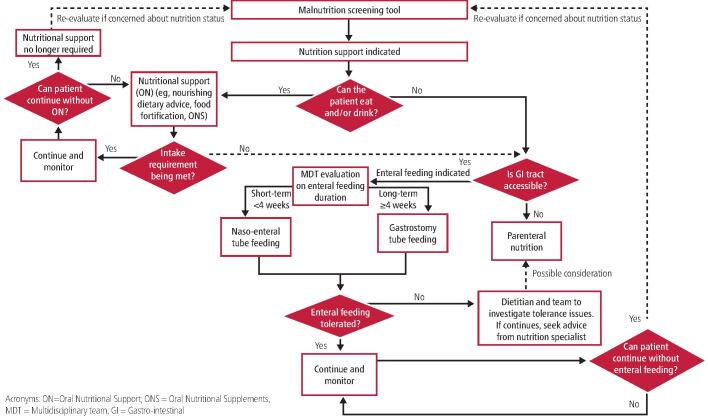
Decision-making for commencing forms of nutrition support

A major role of the dietitian in HNC is initiating, monitoring and reviewing nutritional support requirements. There are three main forms of nutrition support and Figure 2 outlines how decision-making is made:^2^^,^^9^^,^^24^

1. Oral (by assessing current intake and optimising this by offering nourishing dietary advice with or without the use of oral nutritional supplements [ONS])
2. Enteral (by delivery of nutrition via a tube that enters the gastrointestinal tract when oral intake is either contraindicated or insufficient to meet nutritional requirements)
3. Parenteral (by delivery of nutrition via an intravenous route when the gastrointestinal tract is inaccessible or compromised/contraindicated).

Oral nutrition support (ON) is first line practice to prevent further nutritional decline and/or meet existing deficiencies if the oral route is not contraindicated (for example, nil by mouth post surgery or dysphagia). Interventions include providing food fortification, nourishing dietary advice and use of ONS. ONS are high protein/energy formulas, available as liquid, ready-to-drink bottles or powdered to make up with milk or water.^2^ The benefits include the energy-dense formula (as high as 4 kcal/ml), of which most are fortified with vitamins and minerals, making them 'nutritionally complete' in specified volumes, alongside their convenience and wide product range available on prescription. Due to the nature of disease location and side effects of treatment, meeting nutritional requirements via a food-first approach only is often unfeasible. During these periods, energy-dense ONS taken alongside are an effective means to supplement energy/protein deficits.^9^

A randomised controlled trial investigated the impact of use of ONS in HNC patients undergoing radiotherapy or radiotherapy plus systemic treatment and receiving nutritional counselling. It was reported that the use of ONS resulted in a statistically significant smaller loss of body weight, higher protein-calorie intake, improved treatment tolerance and improved QofL compared to nutritional counselling alone.^31^

The disadvantages of ONS include that the majority of products available are sweet flavour options (with limited savoury options available), thus can be high in sucrose and highly cariogenic.^32^ The volume and frequency of supplements therefore needs to be monitored alongside appropriate preventative measures, including oral hygiene, dental care professional support and fluoride regimes, and also on patients with poorly-controlled diabetes (Table 2). Nutritional needs are met with frequent consumption of ONS products which are carbohydrate-rich, with some patients drinking these over a protracted period of time: five to seven times daily. This creates an oral environment which encourages plaque build-up, which in turn causes demineralisation of tooth structure and dental caries. In patients with short-term or long-term xerostomia, caries can progress rather fast as these patients also have reduced oral clearance and reduced buffering capacity.^33^ The priority is to ensure optimum nutrition but it is also essential to ensure that the risk of dental caries is reduced by supportive strategies given by the restorative dentist and dental care professionals. On patients receiving high doses of radiotherapy, dental extractions should be avoided, so preventing a deterioration of the dentition is paramount. Additionally, it can be challenging to provide dental restorations for patients with trismus as a side effect of treatment for HNC, so prevention of dental disease is in the patient's best interest.^33^

**Table 2:** Oral risks and mitigation^36^^,^^45^

Dental consideration	Recommendation
Dental caries and periodontal disease risk	Minimising frequency and exposure times of cariogenic food/drink and safe consumption of ONS is desired. In reality, this may not always be achievable due to tolerance issues. Possible strategies to mitigate the effects include: Aim to time ONS with mealtimes if/when feasible Aiming for full ONS volume in one sitting rather than sipping at regular intervals during the day and/or increasing volume taken in each sitting to reduce frequency across the day if tolerated Advocate plaque removal due to 'stickiness' of ONS Prescription of high concentration fluoridated toothpaste: Advise patients to 'spit but not rinse' after brushing teeth Use of fluoridated mouthwash other times than toothbrushing in the post treatment phase Regular reviews with the restorative dentist and dental care professionals Assessing if high cariogenic foods can be replaced with low cariogenic iso-caloric options, such as: Full-fat milk and dairy products especially those that contain probiotics which can reduce Streptococcus mutans, increase saliva pH and promote a higher plaque index^48^ Fortifying meals with additional fat (for example, oil/butter/cream) Consider replacing some ONS with ONS 'shots' which contain less sucrose Using a straw where possible Regular brushing and flossing Take dentures out after every meal to clean them and at night before sleep Encourage plaque removal with toothbrush before ONS consumption and then ~30 minutes after, topical application of toothpaste that contains Recaldent CP-ACP (casein phosphopeptide-amorphous calcium phosphate) or other remineralising agent for patients at risk of caries. Saliva is normally supersaturated with calcium and phosphate which facilitates repair of initial carious lesions. Patients with xerostomia have reduced buffering and remineralisation capacity Rehabilitation services and where possible, joint dietitian/SLT appointments to support patients to come off ONS if/when feasible Early identification by restorative dentist of 'high risk' patients before commencing radiotherapy considering:^49^ Age Sex Number of decayed, missing or filled surfaces Radiation dose, field and technique Encourage cessation and/or reduction of smoking and alcohol
Osteoradionecrosis	Reviewing dental hygiene alongside nutritional assessment when feasible in appointments especially for high-risk patients Early identification by restorative dentist of 'high risk' patients before commencing radiotherapyconsidering the following risk factors:^49^^,^^50^ Smoking status Primary site in the oropharynx Bone surgery before radiotherapy Concurrent chemotherapy Xerostomia presence Dental extraction pre- radiotherapy ≤20 days between dental extraction and commencing radiotherapy (in practice, most restorative dentists aim for a minimum ten days from extractions to starting radiotherapy)^51^ ≥55 gy radiotherapy dose
Xerostomia	Mouthcare Saliva replacement
Trismus	Difficulty with oral access (liaise with SLTs for use of interventions such as jaw-stretching exercises)
Challenges with eating, drinking and chewing caused by dentition issues	Liaise with restorative dentistry regarding the challenges in tolerating oral diet Lack of occluding pairs of teeth (real or prosthetic) Problems with prostheses Delays or lack of access to implant-based rehabilitation (often due to the need to await osseointegration)
Dental extractions	Medications, for example, analgesia to alleviate pain/discomfort with eating and drinking after dental extractions Texture modifications to alleviate discomfort with eating and drinking and reduce chewing burden (for example, nourishing liquids and soft diet) Counselling by restorative dentists due to negative impact on QofL, as this can be detrimental in regards to speaking, eating, socialising and intimacy^52^

Ready-to-drink ONS have been reported to range between 6.6-27.2 g of sugar/serving; powder mixed with 200 ml full-fat milk ranged between 16.4-35 g sugar/serving; and ONS 'shots' ranged between 0-4 g of sugar/serving.^34^ ONS 'shots' can therefore be a good option alongside oral diet/ready-to-drink ONS/powder, as they are lower in sugar. However, they are not generally recommended in volumes aiming to meet a significant proportion of estimated nutritional requirements (≥50%) as they are not nutritionally complete, are often considered less palatable by patients affecting compliance and can have adverse gastrointestinal effects. Malnutrition and dental caries have both been reported to have negative impacts on QofL.^35^ Dietitians can work closely with restorative dentistry (as per Table 2), diabetes clinical nurse specialists and other MDT members to optimise diet and the safe use of ONS alongside dental health and blood glucose control. Evidence for HNC is limited but follows first principles with regards to dental hygiene:^36^

- Caries is caused by the interaction of fermentable carbohydrates and plaque biofilm on a susceptible tooth surface^37^
- Fermentable carbohydrates include sucrose, glucose, lactose and fructose. Sucrose is especially cariogenic due to its effect on plaque biofilm development, as well as being fermentable^38^
- Caries is preventable
- Good oral hygiene is key for caries prevention
- Tailored fluoride regimes are essential on patients with HNC
- Regular reviews with restorative dentists and dental care professionals are essential to intervene at early stages of dental disease
- Treatments for HNC can cause xerostomia. While xerostomia does not cause dental caries *per se,* reduced quality and quantity of saliva reduces the remineralisation potential of early caries lesions.

Enteral nutrition support is often required in HNC. This may be as early as initial presentation due to the impact of disease location affecting swallowing function and/or the ability to take adequate nutrition orally. Some patients may be advised to be nil by mouth (NBM) due to aspiration risk by SLT and thus will need immediate placement of an enteral feeding tube. Enteral feeding may be used as the sole source of nutrition (for example, when NBM status) or on a partial basis to supplement oral intake (for example, when able to tolerate some oral diet/fluids but inadequate to meet full nutritional requirements).^9^^,^^24^

**Fig. 3:**
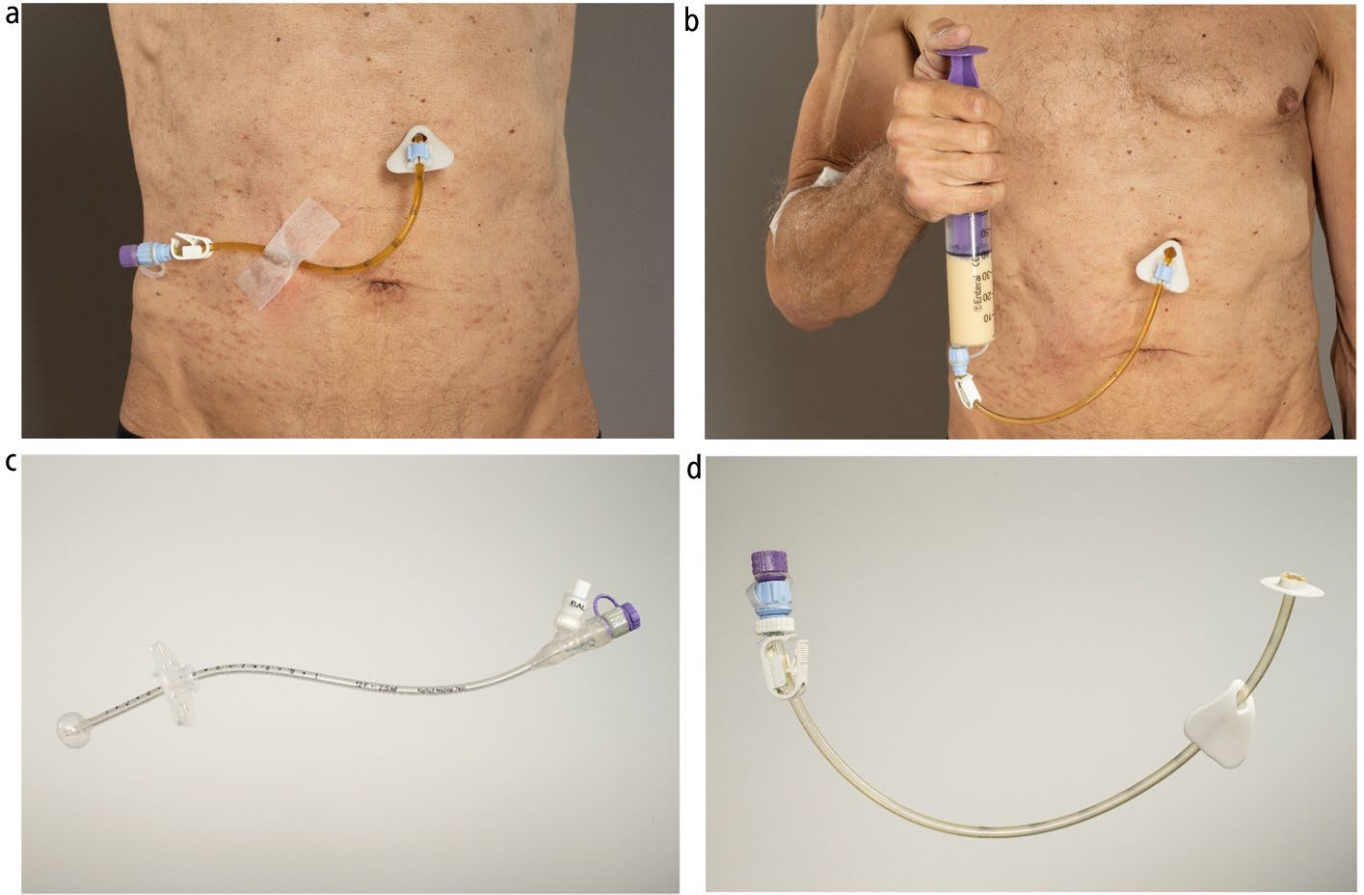
Clinical photographs of a PEG tube. a) Depicts a patient's abdominal site with a 15 French Freka PEG tube (Fresenius Kabi) *in situ *which was placed endoscopically. b) Depicts a patient administering feed through his 15 French Freka PEG tube (Fresenius Kabi) by the 'bolus method'. A syringe is used to draw enteral feed/oral nutritional supplements and then screwed onto the end of the PEG. The PEG tube is unclamped and feed is delivered. c, d) Depicts a demonstration model of a 15 French Freka PEG tube (Fresenius Kabi). The end of the tube has a cap and a clamp is placed on the tube lumen to prevent leakage. An external triangular fixator plate is clamped close to the skin to keep the PEG in position. The tube is retained by an internal bumper/disc. The patient is advised to advance and rotate the tube weekly after the stoma tract has developed (10-14 days post insertion) to prevent 'buried bumper syndrome' (when the internal bumper becomes buried within the wall of the abdomen)

**Fig. 4:**
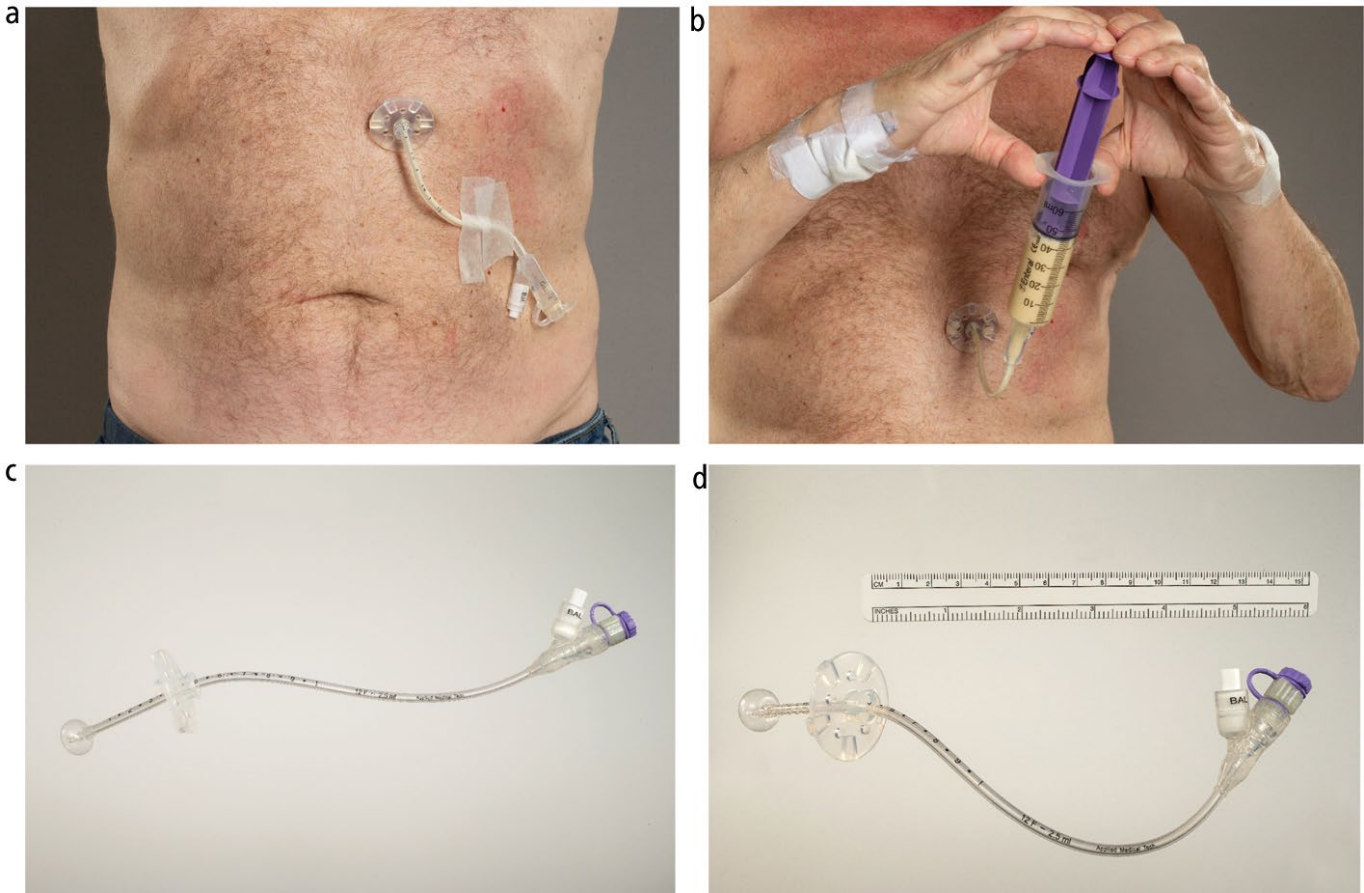
Clinical photograph of a RIG tube. a) Depicts a patient's abdominal site with a 14 French MIC* gastrostomy feeding tube (Avanos) *in situ* which was placed radiologically. b) Depicts a patient administering feed through his 14 French MIC* gastrostomy feeding tube (Avanos) by the 'bolus method'. A syringe is used to draw enteral feed/oral nutritional supplements and then screwed onto the end of the RIG to deliver the feed. c, d) Depicts a demonstration model of a 12 French MIC* gastrostomy feeding tube (Avanos). The end of the tube has a feeding port and a luer lock balloon inflation valve. The feeding port is capped to prevent leakage. An external retention ring is kept close to the skin to keep the RIG in position. The tube is retained by an internal silicone balloon. The balloon is filled with water to keep the tube *in situ*. Once the tract is developed two weeks post RIG insertion, the patient is then advised to change the water weekly thereafter

Enteral feeding tube options include those for the short-term (generally considered <4 weeks but not limited to this time frame) and long-term (≥4 weeks).^2^ Short-term options include naso-enteral tubes such as naso-gastric, naso-jejunal, oesophageal-fistulae and orogastric. Long-term options include gastrostomy tube feeding, such as percutaneous endoscopic gastrostomy (PEG) (see Figure 3), radiologically inserted gastrostomy (RIG) (see Figure 4) or surgical gastrostomy/jejunostomy. Decision-making for the type of tube placed should account for the anticipated length of enteral feeding (with consideration to tumour type, size, location and treatment plan), patient choice and local resources/policy.^24^ In some centres, gastrostomies may be placed before commencing HNC treatment if it is anticipated that treatment may result in requiring enteral feeding for a long-term period. Controversy exists for placement of prophylactic gastrostomy before radiotherapy/chemoradiotherapy and the evidence base remains inconclusive.^39^^,^^40^ Guidelines have been developed to support appropriate decision-making, which often include tumour stage, site, age, nutritional status, pre-existing dysphagia, performance status and impact of planned treatment.^7^^,^^24^^,^^41^^,^^42^ Dietitians are key stakeholders for supporting appropriate decision-making within the MDT, which has led to the development of dietetic-led gastrostomy services in some centres. This enables dietitians to lead on screening, assessing and counselling patients to support appropriate decision-making for gastrostomy insertion and removal, working alongside gastroenterology and radiology departments and HNC MDT members.^43^

Parenteral nutrition is seldom used in HNC as most patients have an accessible gastrointestinal tract and can tolerate enteral nutrition. However, it should be considered when clinically appropriate and in consultation with specialist gastroenterology nutrition teams in hospital settings.

## Consideration of dental care alongside nutritional intervention across the pathway

Dental condition is a risk factor for weight loss in HNC at the outset.^44^ Dietitians work alongside restorative dentists and the wider MDT to ensure nutritional needs are met while balancing the importance of oral hygiene and prevention of dental caries.^33^ This is further explained by the risks outlined in Table 2 and strategies to mitigate these.^36^^,^^45^

## Conclusion

The aetiology of HNC itself, and treatments, can lead to altered anatomy and impaired swallowing function which can compromise nutritional status. Many patients are at risk of malnutrition and require nutritional support. Dietitians have a key role in assessing and treating malnutrition with the provision of nutrition support. Due to the limitations on texture and tolerance of oral diet, use of ONS, nourishing dietary advice and enteral feeding are often required. Dietitians can work with restorative dentists to promote safe consumption of ONS and other high-cariogenic food/drink and in addition, can help with rehabilitation back to a baseline diet alongside SLT when feasible, to reduce reliance on ONS.
